# Personalized Health Care as a Pathway for the Adoption of Genomic Medicine 

**DOI:** 10.3390/jpm2040232

**Published:** 2012-11-13

**Authors:** Robin Burnette, Leigh Ann Simmons, Ralph Snyderman

**Affiliations:** 1 Duke Center for Research on Prospective Health Care, Duke University Medical Center, Durham 27701, NC, USA; E-Mails: robin.burnette@duke.edu (R.B.); leighann.simmons@duke.edu (L.A.S.); 2 Department of Community and Family Medicine, Duke University Medical Center, Durham 27701, NC, USA; 3 School of Nursing, Duke University Medical Center, Durham 27701, NC, USA; 4 Department of Medicine, Duke University Medical Center, Durham 27701, NC, USA

**Keywords:** personalized health plan, multi-morbidities, preventive, health risk assessment, chronic disease management, personalized medicine tools, genomic medicine

## Abstract

While the full promise of genomic medicine may be many years in the future, personalized health care (PHC) can begin solving important health care needs now and provide a framework for the adoption of genomic technologies as they are validated. PHC is a strategic approach to medicine that is individualized, predictive, preventive, and involves intense patient engagement. There is great need for more effective models of care as nearly half of Medicare patients age 65 and older have three or more preventable chronic conditions and account for 89% of Medicare’s growing expenditures. With its focus on reactive care, the current health care system is not designed to effectively prevent disease nor manage patients with multiple chronic conditions. PHC may be a solution for improving care for this population and therefore has been adopted as the delivery platform along with a new personalized health plan tool for 230 multi-morbid, homebound Medicare recipients in Durham, North Carolina who have been high utilizers of health care resources. PHC integrates available personalized health technologies, standards of care, and personalized health planning to serve as a model for rational health care delivery. Importantly, the PHC model of care will serve as a market for emerging predictive and personalized technologies to foster genomic medicine.

## 1. Introduction

The complete sequencing of the human genome in 2000 along with the rapid development of proteomic and related ‘*omic*’ technologies gave rise to the anticipation that genomic-based personalized care would soon transform medicine [1]. Indeed, the Chair’s address, given by one of the authors (RS), to the Association of American Medical Colleges in 2002, detailed the impending transformation of medicine as a consequence of predictive technologies and prospective approaches to care [2]*.* Now, over a decade later, while important personalized medicine tools have been introduced, the basic approach to care remains unchanged and the promise of genomic medicine is anticipated to be many years in the future [3]. Yet, the need for transformation of care is greater than before. In the United States and worldwide, health care systems focus on the reactive treatment of disease events associated with the later stages of chronic, often multi-morbid diseases (the presence of two or more disorders) rendering care inefficient and extremely costly [4,5,6]. As rates of preventable chronic disease and multi-morbidity have increased over the last decade, health care providers have yet to effectively address disease prevention, let alone the personalized needs of individuals managing multiple chronic diseases. This has resulted in increasing expenditures, especially for Medicare patients [5] whose enrollment is anticipated to double over the next 20 years [7]. Unless the current approach changes, the burden on the health care system will be overwhelming [4,8]. The Centers for Medicare and Medicaid Services have recognized the need for a new approach to solve this dilemma and have endorsed personalized and preventive care initiatives to reduce escalating costs to the health care system [9,10]. 

While the promise of genomics remains to be fulfilled, the concepts underlying personalized, prospective health care can be adopted now with available technologies and thereby create a market for emerging genomic technologies [2,10]. Personalized health care (PHC) has been proposed as a rational approach to fill the need for an innovative, cost-effective model for health care delivery [2,8,10,11,12,13]. PHC is a coordinated, strategic approach to patient care that broadly applies the concepts of systems biology and personalized, predictive, preventive, and participatory care (known as prospective health care or P4 medicine) [2,10,11,12,13,14,15,16], and uses available technologies to customize care across the health continuum from health promotion and prevention, to detection and treatment of disease [17]. While PHC anticipates using the vastly improved predictive tools created as a consequence of genomic technologies, it does not require them to get started. PHC is designed to proactively change the trajectory of disease development by using available predictive health risk assessment capabilities and planning while intensively engaging the patient in coordinated approaches to their care. Although personalized medicine is often equated with genomic medicine, PHC, as defined herein, takes a broader view and embraces current capabilities and tools to provide the best strategically planned predictive care [10,17]. Nonetheless, the PHC approach provides a portal for the clinical adoption of genomic technologies as they are validated.

## 2. Personalized Health Care and the Personalized Health Plan

At the heart of PHC is the personalized health plan (PHP), which integrates conventional clinical care with personalized diagnostic and therapeutic capabilities to enable health risk assessment, personalized therapies where available, and outcome tracking to determine progress. Patient engagement is enhanced through intense education to increase patients’ understanding of both their risks and the need for their involvement in setting therapeutic goals. Therefore, the PHP serves as an organizational tool that anticipates health care risks and needs over a defined interval and functions as a point of care coordination between the patient and health providers [10]. The PHP is designed to identify, quantify, and evaluate health risks and opportunities to avoid disease events, slow disease progression, and improve the patient’s health over the time of the plan. Patient engagement and buy-in are essential in determining goals, plan execution, and outcome tracking—the latter of which is a core element of PHC and particularly important for patients with multi-morbidity. The PHP supports patient engagement and provider-patient collaboration to motivate changes in health behavior. The critical elements of the PHP are [13]:

(a) Evaluation of the patient’s current health status to quantify their health needs and risks using the best available predictive tools and clinical judgment. This identifies the patient’s specific health risks and needs over the planned interval of care.
(b) A therapeutic plan designed as an actionable coordinated care plan for a defined period to mitigate risks and improve health. The plan is developed based on clinical judgment, consensus guidelines, outcome algorithms, validated predictive tools, and patient-provider discussions. The plan establishes specific goals both for the patient and provider over the time of the plan.
(c) Tracking disease development or improvement based on measurement of biomarkers, risk factors, and clinical indicators. Depending on the progress of the tracking factors, the plan is refined during the course of care. Feedback between patient and provider are used to encourage adherence with the plan.

The PHP framework provides an excellent operational platform for the delivery of PHC to individuals with diverse needs ranging from those undergoing their annual medical visit to health disparate populations with multi-morbidities. Additionally, risk assessment tools can be utilized to discriminate amongst phenotypes of diseases. For example, in obesity, metabolic profiling can be utilized to mechanistically distinguish the underlying nature of the disorder and thereby customize therapy [18]. For this reason, PHC will be utilized for the care of a population of lower income, predominantly Medicare patients in Durham, North Carolina who are enrolled in a patient-centered medical home program called *Just for Us* (JFU). The JFU program provides in-home primary care to seniors and disabled adults who have physical or psychosocial impediments [19] such as frailty or reliance on public transportation that limit their ability to access care. The JFU program was created in response to the need for coordination among health-associated agencies to reduce gaps in support services. Before the JFU program, many of these vulnerable adults routinely used ambulance services and emergency departments to address their medical needs. Currently, the JFU clinical team (physician, social worker, advance practice provider, staff specialist, occupational therapist, and pharmacist) provides in-home care and case management to approximately 230 seniors and disabled adults who live independently. Eighty-one percent of the enrollees are African American, 63% are women, the average age is 71 years old, and more than 50% are eligible for both Medicare and Medicaid. The top five chronic conditions are hypertension, hyperlipidemia, diabetes, osteoarthritis, and obesity. Approximately 72% have three to seven chronic conditions with almost 10% having eight or more. On average, the JFU patient attempts to manage a complicated regimen of 14 medications with 36% of patients on 16 to 30 medications, which are usually prescribed by different providers. Although JFU endeavors to deliver coordinated health care services, it has been reactive and disease-centric [5]. Over a 12-month period, nearly 26% of JFU patients had emergency room visits with 17.3% being hospitalized. The major reasons for emergency room use were reports of pain, often from a fall, cardiac conditions, and urinary problems. This highlights the need and opportunity to have a plan in place to coordinate care and reduce adverse events. 

**Figure 1:**
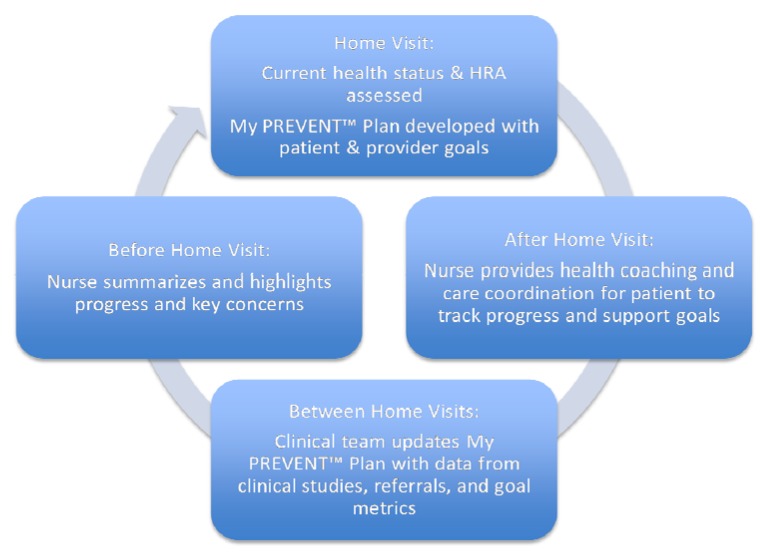
The cyclical work flow from initial Just for Us (JFU) home visit through activities after the visit to preparation for the next home visit.

To implement PHC into the JFU program, a PHP application, My Personalized Risk EValuation, ENgagement, and Tracking Plan (My PREVENT™ Plan) has been developed. The primary aim of My PREVENT™ Plan is the identification of the patient’s proximate and longer-term health risks and needs, chronic disease management, patient engagement, and coordination of care. The plan will embrace existing and new technological advances in health risk assessment as well as personalized therapeutics, where applicable. For each patient, a My PREVENT™ Plan will be developed collaboratively between the patient and JFU provider(s). The plan is based on a comprehensive health profile that determines current health status of the individual. This assessment includes personal history, family history, results from health promotion and disease prevention screens, physical exam, evaluation of functional status and health behavior along with risk assessment tools that add predictive capabilities of clinical value. The plan specifically includes: (a) short-, intermediate- and long-term evidence-based health risk assessment and, when appropriate, individual risk tools, tests and technologies for prediction of disease or clinical events (b) 3-, 6-, and 12-month goals that are established collaboratively between the patient and provider and then tracked, (c) a therapeutic plan to meet the goals, and (d) ongoing coordinated support and health coaching for patient engagement and health plan implementation (Figure 1). Patients will receive a copy of the therapeutic plan organized in 3-month intervals with patient and provider shared goals converted into action steps which will be supplied with the help of a nurse/health coach. Outcomes will be tracked to determine if goals are met. 

Risk assessment, which can include the use of risk assessment tools, is generally an integral yet informal activity in conventional patient care [20]. My PREVENT™ Plan includes evaluation of medical history, family history, psychosocial and environmental issues, formalizes and quantifies this process with risk assessment tools (cardiovascular, metabolic, musculoskeletal, functional ability and health behavior) and biomarkers to create a more robust individual health risk assessment. Predictive and personalized medicine embraces, but is not dependent on, the use of genomics as there are useful health risk assessment tools that have no “-omic” technology [10]. In selecting risk predictive tools for the assessment process, pertinent elements are:

(1) applicability to the target population’s demographics such as age, sex, and prevalence of risk;
(2) validation in large cohorts for greater precision in the estimation of risk and stratification;
(3) clinical usefulness by adding value (not duplication) as an objective measure to the clinician’s judgment and decisions;
(4) production of results that can be used in discussions with the patient to educate and help them make informed decisions about their care and therapy goals;
(5) cost-effectiveness or reimbursable for the health system and the patient; and,
(6) endorsement by experts, other clinical providers, and supported by peer-reviewed publications.

These aspects were taken into account for the initial selection of the risk assessment tools for My PREVENT™ Plan along with reviewing the extant literature to compile a list of over 200 risk assessment tools and tests. The tools were then categorized by system (e.g., metabolic, cardiovascular, *etc*.) and those that provided the most robust validated estimates were identified. To narrow the list for the greatest clinical efficiency, the Pareto principle was employed to identify the disorders that account for 80% of health problems in JFU patients and found that hypertension and diabetes were the leading disorders affecting 87.2% and 50.4% of patients, respectively. Risk prediction tools were selected based on their ability to identify meaningful significant clinical events that occur (e.g., stroke, fracture from falls) in the JFU population. Lastly, the initial risk assessment included tools that had open access with minimal added cost. Clearly, the use of some predictive tools that determine a 10-year risk will be less valuable for a patient with less than a 10-year life expectancy in the JFU population. My PREVENT™ Plan will allow the clinician to choose predictive tools relevant to the individual patient by utilizing drop-down menu selection for relevant risk calculators based on the identified risk factors. 

Given that My PREVENT™ Plan will be implemented via an electronic medical record, data will be automatically populated from the health history and physical exam into the risk assessment tools embedded within the plan application to rapidly produce the quantification of disease burden or risk score. The initial strategy is to start with a focused set of tools and tests (Table 1) and over time evaluate their value and incorporate others when indicated. A number of marketed personalized medicine tools are still under consideration. My PREVENT™ Plan will thus provide a venue for the adoption and/or validation of new predictive tools and personalized technologies within the clinical workflow. 

**Table 1:** Examples of the initial tools selected for health risk assessment and predication for the My PREVENT™ Plan.

**Cardiovascular**	**Indication**
**Framingham CVD Risk Calculator**	Provides a Framingham risk score for a 10-year risk of “hard” CHD outcomes (e.g., MI or cardiac death) in adults ≥20 years old **without heart disease** (e.g., coronary artery disease, valvular disease, arrhythmias, CHF) **or diabetes.**
**Reynolds CVD Risk Calculator (women)**	For women in the intermediate Framingham CVD risk group. This tool can reclassify most of these women into high-risk or low-risk category. Includes the serum CRP and family history into the calculation.
**Serum CRP level**	Serum CRP improves prediction by the Framingham CVD risk score, especially in the categories of intermediate risk males and high risk females.
**Stroke Risk Calculators**	Provides a 10-year risk of stroke. Uses Framingham study cohort of patients 55-84 years old.
	
**Metabolic & Musculoskeletal**	**Indication**
**Diabetes Risk Calculator**	Provides the high, intermediate, or low risk for having pre-diabetes or diabetes using modifiable and non-modifiable risk factors.
**Weight and Health Risk Assessment**	Using BMI, waist circumference and risk factors for diseases and conditions associated with obesity, assesses whether weight loss is needed to lower risk of developing.
**Fracture Risk Assessment (FRAX)**	Provides the 10-year probability of a fracture. Requires the DXA results of the femoral neck BMD (g/cm^2^)
	
**Functional Ability**	**Indication**
**Mini Mental Status Exam** **Clock drawing Assessment**	Cognitive assessment for patients with memory complaints or new functional impairments. Detection of cognitive impairment is part of the Medicare annual wellness visit.
**Timed “Get Up and Go Test”**	A score of ≥3 from this physical exam maneuver indicates an increased risk of falling and need for further evaluation
**Patient Health Questionnaire 2 (PHQ 2) and** **Generalized Anxiety Disorder (GAD-2)**	PHQ 2 screens for depressed mood and anhedonia over a 2-week intervalGAD 2 has high sensitivity and specificity for detecting anxiety disorders.
	
**Health Behavior**	**Indication**
**Patient Activation Measure **	Assessment of confidence & knowledge, staying the course under stress, taking action, and beliefs
**Morisky Medication Adherence Scale **	To measure medication-taking behavior by a 4-item self-report survey

Tools for personalization of care are not only for health risk assessment but also include tools for tracking of outcomes (e.g., biomarkers), evaluation of drug metabolism, and selection of therapy. By using the PHP, one creates a clinical workflow that logically identifies and adopts the best predictive tools to meet the needs of the patient’s plan. As “-omic” technologies are developed to fulfill these needs, they will be incorporated into the PHP. Indeed, there is the intention to communicate with the developers of such tools to pursue potential evaluation for inclusion in the PHP. Given the nature and limitations of reimbursement in the Medicare population and their inability to meet copay perquisites, future personalized tool selections for the JFU population will be largely based on their ability to improve care and reduce cost to the patient. 

The plan has already been pilot tested in eight JFU patients and providers reported that (1) the PHP was completed in the same amount of time as the routine home visit; (2) there was a notable systematic progression of data collection and overall the clinical visit was similar to the traditional clinical note; (3) information gathered was more meaningful and allowed the provider to have a more comprehensive view of the patient’s past and current health status along with social and behavioral determinants and patient preferences; (4) subjective questioning about quality of life was addressed and seemed to increase patient satisfaction and engagement; and (5) patients were enthusiastic and willing to participate in an actual plan of action. 

Cost-savings and cost-effectiveness will be evaluated over the 2-year project with a lifetime sensitivity analysis. There will be particular interest in any change in health care utilization costs (emergency room use and re-admissions) by comparing the health and cost outcomes from JFU patients to matched controls using data from the Centers for Medicare and Medicaid Services. Knowledge gained from the integration of PHC and My PREVENT™ Plan with the JFU program will be translated for broader adaptation within other primary care settings and health care systems. 

The My PREVENT™ Plan as described herein is most applicable to the population of patients with co-morbidities that it was designed to serve. Its basic structure, however, is generally applicable to many other clinical needs including health promotion, primary care, and management of other chronic diseases. For other uses, the basic structure of the My PREVENT™ Plan would stay the same but the risk assessment, tracking, therapeutic tools and plans would change according to the clinical need. Regardless of its application, the My PREVENT™ Plan provides an excellent portal for the adoption of genomic tools as they become validated and demonstrated to add value to the plan. For example, tools for differentiation of disease mechanism amongst phenotypes, genomic-based health risk assessments, drug metabolic indicators or targeted therapy will find logical points of entry in My PREVENT™ Plans whenever they are appropriate. Thus, the approach described here, while not dependent on new technologies, provides a logical and valuable clinical platform for their adoption. 

## 3. Conclusions

As health care systems face the challenge of an increasing aging population with complex multi-morbid conditions and the escalation of medical costs, a new model of care is needed. PHC using My PREVENT™ plan incorporates the principles of personalized, predictive, preventive, participatory, and prospective care into a working model. Integration of appropriate personalized health technologies, standards of care, and health planning into My PREVENT™ Plan not only enables a rational, strategic approach to care, but also creates a means to identify and effectively utilize emerging personalized medicine technologies to improve care. While the promise of genomics technologies may be decades from their full benefit to medicine, PHC is ready now. Its adoption will not only improve care, it will guide the development of needed personalized medicine tools. 

## References

[1] Williams R.S., Willard H.F., Snyderman R. (2003). Personalized health planning. Science.

[2] Snyderman R., Williams R.S. (2003). Prospective medicine: The next health care transformation. Acad. Med..

[3] Feero W.G., Guttmacher A.E., Collins F.S. (2008). The genome gets personal—Almost. JAMA.

[4] Barnett K., Mercer S.W., Norbury M., Watt G., Wyke S., Guthrie B. (2012). Epidemiology of multimorbidity and implications for health care, research, and medical education: A cross-sectional study. Lancet.

[5] Tinetti M.E., Fried T.R., Boyd C.M. (2012). Designing health care for the most common chronic condition—Multimorbidity. JAMA.

[6] Salisbury C. (2012). Multimorbidity: Redesigning health care for people who use it. Lancet.

[7] Montgomery L. (2011). Good and Bad News for Medicare. The Washington Post.

[8] Dinan M.A., Simmons L.A., Snyderman R. (2010). Commentary: Personalized health planning and the Patient Protection and Affordable Care Act: An opportunity for academic medicine to lead health care reform. Acad. Med..

[9] MLN Matters: Annual Wellness Visit (AWV), Including Personalized Prevention Plan Services (PPPS). http://www.cms.gov/Outreach-and-Education/Medicare-Learning-Network-MLN/MLNMattersArticles/downloads/MM7079.pdf.

[10] Simmons L.A., Dinan M.A., Robinson T.J., Snyderman R. (2012). Personalized medicine is more than genomic medicine: Confusion over terminology impedes progress towards personalized health care. Pers. Med..

[11] Snyderman R., Dinan M.A. (2010). Improving health by taking it personally. JAMA.

[12] Snyderman R., Yoediono Z. (2006). Prospective care: A personalized, preventative approach to medicine. Pharmacogenomics.

[13] Yoediono Z., Snyderman R. (2008). Proposal for a new health record to support personalized, predictive, preventative and participatory medicine. Pers. Med..

[14] Hood L., Balling R., Auffray C. (2012). Revolutionizing medicine in the 21(st) century through systems approaches. Biotechnol. J..

[15] Hood L., Friend S.H. (2011). Predictive, personalized, preventive, participatory (P4) cancer medicine. Nat. Rev. Clin. Oncol..

[16] Smarr L. (2012). Quantifying your body: A how-to guide from a systems biology perspective. Biotechnol. J..

[17] Snyderman R. (2012). Personalized health care: From theory to practice. Biotechnol. J..

[18] Newgard C.B., An J., Bain J.R., Muehlbauer M.J., Stevens R.D., Lien L.F., Haqq A.M., Shah S.H., Arlotto M., Slentz C.A. (2009). A branched-chain amino acid-related metabolic signature that differentiates obese and lean humans and contributes to insulin resistance. Cell Metab..

[19] Yaggy S.D., Michener J.L., Yaggy D., Champagne M.T., Silberberg M., Lyn M., Johnson F., Yarnall K.S.H. (2006). Just for Us: An academic medical center-community partnership to maintain the health of a frail low-income senior population. Gerontologist.

[20] Mark D.B. (1994). An overview of risk assessment in coronary artery disease. Am. J. Cardiol..

